# Comparing TMS perturbations to occipital and parietal cortices in concurrent TMS-fMRI studies—Methodological considerations

**DOI:** 10.1371/journal.pone.0181438

**Published:** 2017-08-02

**Authors:** Joana Leitão, Axel Thielscher, Johannes Tuennerhoff, Uta Noppeney

**Affiliations:** 1 Max Planck Institute for biological Cybernetics, Tübingen, Germany; 2 Computational Neuroscience and Cognitive Robotics Centre, University of Birmingham, Birmingham, United Kingdom; 3 Laboratory for Behavioral Neurology and Imaging of Cognition, Department of Neuroscience, University of Geneva, Geneva, Switzerland; 4 Department of Electrical Engineering, Technical University of Denmark, Lyngby, Denmark; 5 DRCMR, Copenhagen University Hospital Hvidovre, Hvidovre, Denmark; 6 University Clinic of Neurology, Tübingen, Germany; University Medical Center Goettingen, GERMANY

## Abstract

Neglect and hemianopia are two neuropsychological syndromes that are associated with reduced awareness for visual signals in patients’ contralesional hemifield. They offer the unique possibility to dissociate the contributions of retino-geniculate and retino-colliculo circuitries in visual perception. Yet, insights from patient fMRI studies are limited by heterogeneity in lesion location and extent, long-term functional reorganization and behavioural compensation after stroke. Transcranial magnetic stimulation (TMS) has therefore been proposed as a complementary method to investigate the effect of transient perturbations on functional brain organization. This concurrent TMS-fMRI study applied TMS perturbation to occipital and parietal cortices with the aim to ‘mimick’ neglect and hemianopia. Based on the challenges and interpretational limitations of our own study we aim to provide tutorial guidance on how future studies should compare TMS to primary sensory and association areas that are governed by distinct computational principles, neural dynamics and functional architecture.

## Introduction

Neglect and hemianopia are two neuropsychological syndromes that are associated with reduced awareness for visual signals in patients’ contralesional hemifield. While neglect results primarily from right inferior parietal and temporal lesions impairing spatial and temporal attention [1–5], hemianopia is caused by lesions to primary visual cortex leading to selective visual perceptual deficits [6]. Contrasting these two syndromes therefore offers the unique possibility to dissociate retino-geniculate and retino-colliculo circuitries whereby ‘unaware’ visual signals can impact human behaviour [7–9]. However, the insights gained from patient fMRI studies are limited by the fact that lesions are often widespread and heterogeneous potentially affecting underlying white matter tracts resulting in a large variability in behavioural deficits and performance. Further, patients’ symptoms and neural mechanisms may have changed as a result of long-term functional reorganization and compensatory behavioural adaptation after stroke or other permanent focal lesions.

Transcranial magnetic stimulation (TMS) allows one to circumvent these problems by transiently perturbing ongoing activity to a particular task-relevant cortical region under the strict experimental control of the applied TMS protocol. In addition, when combined with functional magnetic resonance imaging (fMRI) it is possible to measure TMS effects not only locally in the stimulated area but also in remote brain areas thereby providing insights into the dynamic interactions between cortical areas [10–14].

We used concurrent TMS-fMRI as a complementary technically challenging transient perturbation approach to compare the functional contributions of parietal and occipital regions to the network of regions involved in visual perception and attention. In a sustained spatial attention paradigm participants had to detect low contrast visual targets that were presented in their left lower visual quadrant on 50% of the trials. The contrast of the target was adjusted individually for each participant to enable approximately 70% detection rate. In two separate sessions, we applied 4 TMS pulses (10 Hz) starting 200 ms after trial begin (i.e. 100 ms after target onset on target present trials) to the right intraparietal sulcus or the right occipital cortex (BA17/BA18) or during a third additional control Sham-TMS session. TMS pulses were applied at intensity that did not significantly affect behavioural performance in any of the three conditions. Critically, while permanent lesions to both primary visual and higher order association areas in parietal cortices are known to result in deficits of perceptual awareness, they are located at distinct cortical hierarchical levels. Hence, comparing the effects of TMS to occipital and parietal cortices on visual processing (i.e. state-dependent TMS effect: interaction between visual input and TMS) allows us to elucidate the underlying neural mechanisms. We expected TMS to occipital cortices to substantially reduce visual evoked responses in lower level visual areas with partially preserved activations in parietal cortices mediated via the intact retino-colliculo circuitry. By contrast, TMS to parietal cortices would predominantly affect visual processing in higher order visual areas.

Starting from the results and challenges of our study this communication will focus predominantly on the methodological and interpretational limitations when comparing TMS effects in primary sensory and higher order association regions that are governed by distinct functional principles. We aim to provide tutorial guidance for future concurrent TMS-fMRI experiments intended to mimick and complement permanent lesion studies in neuropsychological patients.

## Materials and methods

### Participants

Ten right-handed participants (4 male; mean age: 31.5 years; standard deviation: 8.1; Edinburgh Handedness inventory score (mean ± SD) of 78±16.8) with no history of neurological illness, normal or corrected-to-normal vision and reported normal hearing took part in the study after giving written informed consent. Because of technical failure two participants did not participate in the experiments with occipital TMS stimulation. The current comparison between TMS to occipital and parietal cortices therefore focuses on 8 participants that took part in both parts.

The study was approved by the Human Research Ethics Committee of the Medical Faculty at the University of Tübingen. The data from IPS stimulation have been included in a previous report [15].

### Experimental design and task

**Fig 1:**
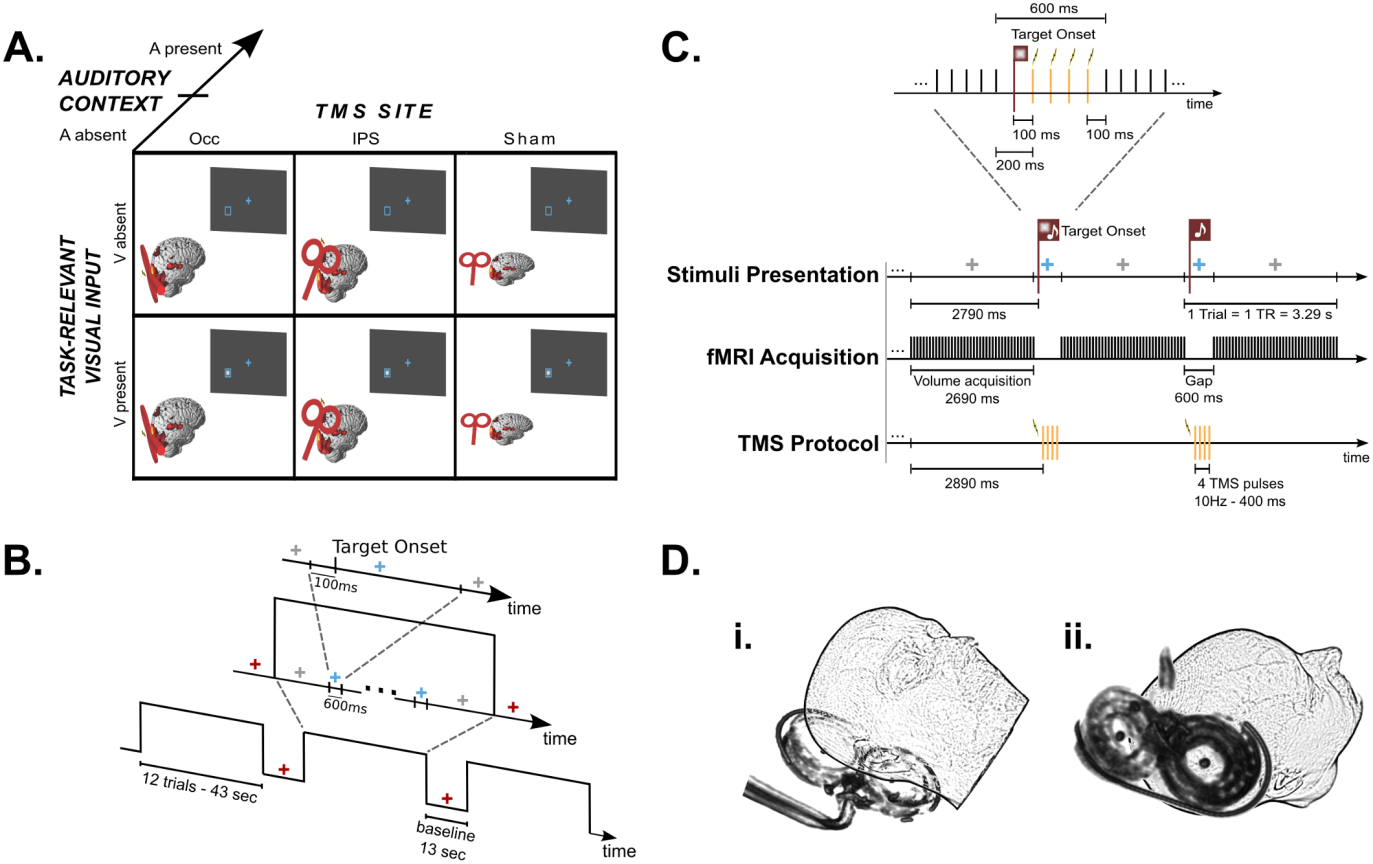
Experimental design. **(A)** 2x2x3 factorial design manipulating (i) task-relevant visual input (V present, V absent), (ii) auditory context (A present, A absent) and (iii) TMS condition (Occ, IPS, Sham). **(B)** Timeline example of stimuli presentation. Blocks of 12 trials started and ended with a grey fixation cross and were interleaved with baseline periods, during which the fixation cross turned red. A trial began when the fixation cross turned blue. In target present trials, the visual stimulus was presented 100 ms after trial begin. After a total period of 600 ms the fixation cross turned back to grey and remained like this until the next trial. **(C)** Illustration of the concurrent TMS-fMRI protocol and stimuli presentation timing during auditory present runs. Within a block the fixation cross was grey during volume acquisition and blue during the acquisition gaps. At 100 ms after trial begin (i.e. 2790 ms after begin of volume acquisition), the task-relevant visual stimulus was either present (first depicted trial) or absent (second depicted trial). Bursts of 4 TMS pulses were applied during acquisiton gaps at 10 Hz and started 100 ms after the target onset time (i.e. 2890 ms after begin of volume acquisition). **(D)** Illustration of approximate coil positions during i) occipital and ii) parietal stimulation.

In a sustained spatial attention paradigm, participants detected visual targets that were presented in a placeholder in the left lower quadrant on 50% of the trials. Across sessions we manipulated whether parietal, occipital or Sham-TMS was applied. Moreover, we also manipulated the presence vs. absence of concurrent task-irrelevant auditory inputs across runs. In short, the paradigm conformed to a 2x2x3 factorial design with factors: (i) task-relevant visual input (V present, V absent) (ii) auditory context (A present, A absent) and (iii) TMS condition (right occipital cortex (Occ), right anterior IPS, Sham) (Fig 1A). Hence, our design included the following 4 trial types: (i) visual target present, without sound (V), (ii) visual target absent, without sound (¬V)), (iii) visual target present with sound (AV) and (iv) visual target absent with sound (A). Each trial type was presented with Occ-, IPS- and Sham-TMS resulting in 12 conditions in total. We limited the presentation of the visual target to the left hemifield because right parietal and occipital TMS has previously been shown to elicit different effects for contra- and ipsi-lateral visual stimuli (e.g. [16, 17, 18]). In all trial types, participants reported whether they had detected the visual target (see *Visual and auditory stimuli)* via a two choice key press. They were instructed to use a strict decision criterion and report that they had detected a target only when being confident and to report ‘unseen’ otherwise. Participants fixated a cross presented in the centre of the screen throughout the entire scanning session.

At the beginning of each trial, the fixation cross changed its colour from grey to blue (Fig 1B). After 100 ms the visual target was presented for a duration of 16 ms with 50% probability. On each trial, we applied bursts of 4 TMS pulses (or Sham-TMS) 200 ms after trial onset (i.e. 100 ms after target onset in V and AV trials) (see *Data acquisition and TMS procedures*; Fig 1C). At 600 ms of trial onset, the fixation cross turned back to grey for a duration of 2690 ms until a change in the colour of the fixation cross indicated the onset of the next trial. The interstimulus interval amounted thus to 3290 ms, equalling one TR of the EPI acquisition (see *Data acquisition and TMS procedures*).

In half of the runs, a sound (see *Visual and auditory stimuli*) was presented synchronously with target onset, regardless of the presence/absence of the task-relevant visual input. Hence, the sound did not predict the presence of the visual stimulus. Nevertheless, the presence of the sounds may have reduced participants’ uncertainty about the temporal onset of the visual target. Auditory sounds were included because it has been shown that the presence of a synchronously presented auditory sound can facilitate the detection of contra-laterally presented visual signals in hemianopia and neglect patients [19]. Yet, the effect of auditory stimuli will not be examined further in this report, which focuses on the methodological aspects of stimulating lower sensory and higher-order association cortices.

Blocks of twelve trials were interleaved with fixation baseline periods of 13 seconds. Each block contained an initial period of 2790 ms to signal the upcoming appearance of the first trial of the block (see Fig 1C). Additionally, to avoid carry-over effects of the response to the last trial into the baseline periods, an extra 3290 ms were introduced at the end of each block. These time lengths were chosen based on the EPI acquisition (see *Data acquisition and TMS procedures*; Fig 1C). Therefore, each block effectively consisted of 13 TRs of the EPI acquisition, resulting in a block length of about 43 seconds. The fixation periods were indicated by a red fixation cross. The beginning and end of the activation blocks were indicated by a grey fixation cross and the detection trials by a blue fixation cross. Hence, the colour of the fixation cross indicated changes in the attentional settings: while blue and grey were associated with a high attentional load, red indicated little attentional demands.

Each run encompassed seven activation blocks with 42 target present trials and 42 target absent trials (i.e. 84 trials per run). The data of the main experiment were acquired in three sessions on different days with each session including eight runs. Across days/sessions, we manipulated whether Occ-, IPS- or Sham-TMS was applied. On each day, we manipulated the auditory context across runs within an ABBAABBA design counterbalanced across participants (i.e. 4 ‘auditory context present (A)’ and 4 ‘auditory context absent (B)’ runs per day). The visual target presence was randomized as trials within and across runs. Hence, we obtained a total of 168 trials for each condition (e.g. number of trials for condition ‘visual target present, auditory context present, IPS-TMS: 42 target-present trials per run x 4 runs with auditory context present x 1 TMS session for IPS). Each participant was trained in a minimum of six runs prior to the actual fMRI experiment.

### Visual and auditory stimuli

#### Visual stimuli

The task-relevant visual stimulus consisted of a small (9x9 pixels, visual angle: 0.52°) square presented for one frame (i.e. 16 ms) on a grey background. The visual stimulus was presented in the centre of a blue placeholder (40x40 pixels, visual angle: 2.3°) that was positioned 12° left and 5° down relative to the fixation cross. The placeholder was displayed throughout the entire run (i.e. including fixation periods).

The ‘overall grey level of the target square’ was adjusted with the help of dithering for each participant in a Quest Procedure [20] inside the scanner aiming at a detection threshold of 70% and using the same parameters as in the main experiment. In other words, the ‘overall grey level’ was adjusted by manipulating the density of white pixels within the square. This detection threshold was selected to place increased demands on cognitive resources such as spatial attention. Importantly, identical grey levels were used across Occ, IPS and Sham stimulation and across auditory contexts.

#### Auditory stimuli

To ensure that auditory stimuli were easily segregated from the scanner noise and TMS clicks we generated an auditory stimulus by adding sinusoidal tones with base frequencies of 130.81 Hz, 164.81 Hz and 196 Hz and the following six terms of their respective geometric progressions (i.e. adding the terms 2^n^*f, where f represents each of the three base frequencies and 1 ≤ n ≤ 6). Hence, the auditory sound spanned a total of seven octaves and ranged from 130.81 Hz to 12543.58 Hz. The duration of each auditory stimulus was 40 ms.

Next, we convolved this auditory signal with spatially specific head-related transfer functions (HRTFs) to create a left localized stimulus. This will provide participants with audiovisual spatial localization cues and thereby enhance audiovisual integration. The HRTFs were pseudo-individualized by matching participants’ head width, height, depth and circumference to the anthropometry of participants in the CIPIC database [21].

### Stimulus presentation

Visual and auditory stimuli were presented using Psychophysics Toolbox version 3.0.10 [22, 23] running on MATLAB 7.9 (MathWorks Inc, MA, USA) and a Macintosh laptop running OS-X 10.6.8 (Apple Inc, CA, USA). The visual stimulus was back-projected onto a frosted Plexiglas screen using a LCD projector (JVC Ltd., Yokohama, Japan; resolution: 800x600 pixels, refresh rate: 60 Hz, viewing distance: 48 cm) visible to the participant through a mirror mounted on the MR head coil. Auditory stimuli were presented via MR-compatible electrodynamic headphones at a sampling frequency of 44100 Hz (MR Confon GmbH). Furthermore, earplugs were used to attenuate both scanner and TMS noise.

Participants indicated their response (i.e. visual target seen or unseen) with their right hand using an MR-compatible custom-built button device connected to the stimulus computer.

### TMS sites

TMS was applied over the right anterior IPS and the right occipital cortex (Occ) as experimental sites and Sham TMS was included as a control condition.

For the parietal stimulation site, we selected the MNI coordinates (x = 42.3, y = -50.3, z = 64.4) that had previously been associated with impairment of visuospatial processing by Oliver et al [24].

For the occipital stimulation site, we determined the MNI coordinates in an initial pilot hunting procedure outside the MRI scanner in seven additional participants. The hunting procedure used the same visual display and task as in the main experiment, established protocols for visual suppression (i.e. single TMS pulse applied 100 ms after target onset; [25]) and a 3x3 grid of coil positions with an equidistant spacing of 1 cm, placed over the right hemisphere guided by Thielscher et al. [26]. The positions showing larger decrements in detection performance across participants were located in the middle row of the grid (hit rates (± SD) from left to right were 41 ± 26%, 43 ± 26% and 42 ±16%, respectively). As the final location for occipital TMS stimulation we selected the MNI coordinates that were associated with the maximal decrement in detection performance across participants (MNI coordinates: x = 19.42, y = -102.35, z = 13.4). Based on cytoarchitectonic probabilistic mapping [27] this position is located in BA17/18.

With the bursts of 4 pulses and the stimulation intensity used in the TMS-fMRI experiment (see *Data acquisition and TMS procedures*), this position did not induce peripheral nerve stimulation that could cause discomfort to the participants. However, occipital stimulation inside the scanner inevitably involves lying on the TMS coil, which is associated with additional discomfort.

Individual stimulation coordinates for both experimental TMS sites were determined by inverse transforming the MNI coordinates for parietal and occipital targets into native space using the parameters obtained from spatial normalization. These coordinates were entered in the neuronavigation system, which was used to mark the desired position on the participants’ skull. For occipital stimulation, the coil was oriented so that the electric field in the stimulation hotspot was oriented from lateral to medial for the second half of the biphasic stimuli. For IPS stimulation the coil was oriented so that the current flow was from anteromedial to posterolateral in the second half of the stimulus (Fig 1D).

*A posteriori* coil reconstruction of the coil position was based on custom-written MATLAB (MathWorks Inc, MA, USA) scripts. The centre of the TMS coil and its circumference were marked with Vitamin E capsules and a water tube, respectively, to enable the automatic co-registration of the coil representation in the FLASH images with a pre-acquired reference image of the coil. The coil representation included a line passing perpendicularly (to coil surface) through the centre of the coil, which allowed determining the coordinates where it first touched the cortical surface. In addition, the subject’s head in the FLASH images was co-registered to the high-resolution structural scan. Thereby, we were able to determine the coil position inside the scanner with respect to an individual’s structural MRI. Across participants, the target IPS coordinates were obtained with a mean deviance of 9 mm ± 2.5 (mean, SD). The across-participants mean coordinate in MNI space was (x = 34.7, y = -52.8, z = 63.5). The across-participants mean of the target Occ coordinates in MNI space was (x = 18.8, y = -100.9, z = 11.4) with a mean deviance of 5.1 mm ± 1.7 (mean, SD). Due to the quadratic decay of the TMS-induced magnetic field and the use of a fixed stimulation intensity across all participants and stimulation sites (see *Data acquisition and TMS procedures*), it is important to make sure that coil-cortex distances do not significantly differ between the two TMS conditions, as these could confound the comparisons of the TMS-induced cortical effects between the two sites. For each participant and each stimulation location, we calculated the Euclidean distance between the centre of the coil and the reached cortical coordinates (i.e. the location where the normal to the coil intersects with the brain surface). A paired t-test showed that the distances between coil and this location defined on the brain surface did not differ significantly for the two stimulation sites (*t*_*(8)*_ = -1.764, *p* = 0.12). These results suggest that differences in TMS effects for the different TMS sites cannot be explained by differences in the coil-brain surface distances across participants.

In the sham condition, 2 cm thick plastic plates were fixed between the TMS coil and the skull. Given the quadratic decay of the TMS-induced magnetic field, this Sham condition precluded the effects of direct brain stimulation. Indeed, when tested over the finger region of the motor cortex, this Sham condition did not induce muscular twitches on pre-activated finger muscles even at 100% of total output intensity. During the Sham condition the coil was placed over the right hemisphere in a middle position between experimental locations, given the space constraints inside the MR coil. Critically, the Sham-TMS condition tightly controlled for the TMS side effects such as the TMS-noise and feelings of vibrations. Furthermore, comparing IPS-TMS or Occ-TMS with Sham-TMS did not elicit significant activations in the auditory cortex. Hence, we concluded that this particular application of Sham-TMS inside the scanner is a better control than low intensity TMS that does not control effectively for auditory confounds (see *Data acquisition and TMS procedures* and [14]).

### Data acquisition and TMS procedures

A 3T TIM Trio System (Siemens, Erlangen, Germany) was used to acquire both a T1-weighted three-dimensional high-resolution structural image (MPRAGE, 176 sagittal slices, TR = 2300 ms, TE = 2.98 ms, TI = 1100 ms, flip angle = 9°, FOV = 240 mm x 256 mm, image matrix = 240 x 256, voxel size = 1 mm x 1 mm x 1 mm, using a 12-channel head coil) and T2*-weighted axial echoplanar images (EPI) with blood oxygenation level dependent (BOLD) contrast (GE-EPI, TR = 3290 ms, TE = 35 ms, flip angle = 90°, FOV = 192 mm x 192 mm, image matrix 64 x 64, 40 axial slices acquired sequentially in ascending direction, slice thickness = 3 mm, interslice gap = 0.3 mm, voxel size = 3 mm x 3 mm x 3.3 mm, using a 1-channel Tx/Rx head coil). Each participant took part in a total of eight experimental runs per TMS condition. A total of 124 volume images were acquired for each run.

After each EPI run, a fast structural image (fast low-angle shot [FLASH], 100 axial slices, TR = 564 ms, TE = 2.46 ms, FOV = 256mm x 256 mm, image matrix = 256x256, voxel size = 1x1x3 mm) was acquired to enable *a posteriori* reconstruction of the TMS coil position inside the scanner, as described elsewhere [14].

The EPI sequence was adapted for concurrent TMS-fMRI experiments by introducing gaps of 600 ms after every volume acquisition. Each gap was introduced to allow the delivery of four TMS pulses without interference with image quality [28, 29]. While this fixed relationship between TMS and MR acquisition does not enable continuous sampling of the hemodynamic response function, it is a common practice in concurrent TMS-fMRI study to minimize image artefacts caused by the application of a TMS pulse. Bursts of four pulses at 10 Hz were applied every trial, with the first pulse applied 2890 ms after begin of volume acquisition, i.e., 100 ms after stimulus onset (Fig 1C). TMS pulses were applied after stimulus onset in order to minimize crossmodal interaction effects between our stimuli and the TMS induced auditory and somatosensory side effects [14, 30].

Similar TMS protocols have been used over the parietal cortex in TMS studies outside the scanner [24, 31] and in concurrent TMS-fMRI studies [10–12, 32–34] investigating the effects of parietal TMS on visuospatial processing. However, occipital TMS studies have mainly been performed using a single or double pulse TMS, in which an appropriate timing relative to stimulus onset (effective time window: 80–110 ms) is crucial in the generation of suppression effects [35, 36]. To allow for comparison between parietal and occipital TMS conditions, we also applied bursts of 4 TMS pulses in the occipital stimulation with the first pulse being applied within the classical effective suppression time window and the remaining three pulses in a wider window that may affect higher order visual processing (see [25]). Likewise, the TMS intensities of each pulse were the same (see below) across parietal and occipital stimulation conditions. Consequently, the intensity of the occipital stimulation was most likely in a range where it perturbs activity locally under the coil and in remote brain areas as well as effective connectivity between brain areas rather than actively impairing visual detection performance. Indeed, previous studies have shown that visual evoked potentials can be influenced at TMS intensities that do not yet affect participants’ visual discrimination performance (e.g. [37]).

Biphasic stimuli were delivered using a MagPro X100 stimulator (MagVenture, Denmark) and a MR-compatible figure of eight TMS coil (MRi-B88), using the same coil-holding device as described in Moisa et al [29]. To prevent the propagation of RF noise into the MR room, the TMS stimulator was placed outside the MR room and was connect to the TMS coil via a high-current filter (E-LMF-4071; ETS- Lindgren, St. Louis, MO, USA) attached to the copper shielding of the MR room [29]. During occipital stimulation the coil was directly placed on a cushion in the RF coil with the major axis oriented parallel to the scanner bore axis and the cable pointing to the right relative to participants’ heads ([38] and Fig 1D).

During IPS and Occ stimulation, a fixed TMS intensity of 69% of total stimulator output was used for all participants. This corresponded to 125% of the mean resting motor threshold, as determined across twenty-four participants of prior studies using the same coil. To ensure similar somatosensory side effects between experimental conditions and Sham-TMS the TMS intensity was increased to 75% of total stimulator output during the Sham condition based on the subjective report of two naïve participants that participated in a pilot test.

Extensive image quality tests of our setup are reported elsewhere ([29], [39]: Supplementary Material). For completeness, we acquired EPI data with a phantom using the same experimental design. After realignment, data were entered in a first level analysis using the same model as for the real participants. Computing all the relevant contrasts (height threshold: *p* < 0.01 uncorrected) yielded only a spurious and randomly distributed pattern.

### fMRI data analysis

The fMRI data were analysed using SPM8 (Wellcome Department of Imaging Neuroscience, London; www.fil.ion.ucl.ac.uk/spm) [40]. Scans from each participant were realigned using the first as a reference, unwarped, spatially normalized into MNI space, resampled to a spatial resolution of 2 x 2 x 2 mm^3^, and spatially smoothed with a Gaussian kernel of 8 mm full-width at half-maximum. The time series of all voxels were high-pass filtered to 1/128 Hz. The first 3 volumes were discarded to allow for T1-equilibration effects.

The fMRI experiment was modeled as a mixed block-event-related design. Individual trials were modeled as events and entered into a design matrix after convolution with a canonical hemodynamic function and its first temporal derivative. Each run included separate regressors for visual present vs. visual absent trials as two types of events. In addition to modeling these two trial types, our statistical model modeled block begin and end (i.e. the periods during which the fixation cross was grey at the beginning and end of a block; see *Experimental design and task*) as mini blocks of 2.69 s and 3.29 s duration, respectively. These additional regressors were included to explicitly account for the increased attentional demands at the beginning and end of the task blocks (note that models that did not include these additional regressors provided basically equivalent results for contrasts combining the remaining regressors). To allow for a more efficient estimation we concatenated the four runs for each of the 3 (Occ, IPS, Sham-TMS) x 2 (auditory present vs. absent) combinations and modeled the run-specific means as separate regressors. Hence, the factors of auditory context and TMS were manipulated across runs and sessions, respectively. Nuisance covariates included the realignment parameters to account for residual motion artifacts.

For each participant, condition specific effects were estimated according to the general linear model by creating contrast images of each condition (i.e. limited to the canonical hemodynamic response) relative to the arbitrary baseline. Hence, the baseline controls for non-specific effects caused by the positioning of the coil (e.g. discomfort during the session with occipital TMS). The statistical comparisons (see details listed below) were entered into independent second-level one-sample t-tests to allow for random effects analyses and inferences at the population level [41].

For full characterization of the data, we report activations at the cluster level at *p* < 0.1 corrected for multiple comparisons (family-wise error rate) within the entire brain based on non-parametric permutation testing [42, 43] using an auxiliary uncorrected voxel threshold of *p* = 0.01. However, we only discuss activations that are significant at p<0.05 corrected for multiple comparisons in the entire brain. Moreover, based on our apriori hypothesis and questions we report activations at the voxel level corrected for multiple comparisons within right IPS and right lower level visual areas (i.e. V1 + V2) as our regions of interest (ROI) where Occ and IPS-TMS were applied. The regions of interest were defined based on cytoarchitectonic probabilistic mapping [27]. Right IPS combined right hIP1, hIP2 and hIP3, while low level right visual regions included right hOC1 and hOC2.

#### Main effects of task-relevant visual input

Main effects of task-relevant visual input were tested for by comparing visual target present (= AV + V) and visual target absent trials (= A + ¬V) pooled (i.e. summed) over TMS conditions (i.e. we performed the comparisons “target present > target absent” and “target absent > target present” pooled over the remaining factors).

#### Main effects of TMS

Since TMS effects in the absence of a neutral control condition may result in interpretational ambiguities, comparisons between TMS conditions were implemented in a paired-wise fashion by comparing each experimental condition (IPS or Occ) with the control condition (Sham). While not the main focus of this study, comparisons between each experimental condition with each other were also computed for completeness. Hence, effects of IPS-TMS were identified by comparing IPS > Sham and Sham > IPS pooled (i.e. summed) over conditions. Equivalent contrasts were calculated for Occ-TMS and for direct comparisons between the two experimental conditions.

Please note that formally these ‘main effects of TMS’ test for an interaction (e.g. main effect of Occ-TMS > Sham can be more precisely written as: (TMS stimulation—Baseline) under Occ-TMS > (TMS stimulation—Baseline) under Sham-TMS). Hence, this interaction contrast controls for non-specific TMS effects such as discomfort that may have been increased for Occ-TMS.

#### State-dependent TMS effects: Interaction effects between visual input and TMS

Likewise, interaction effects between task-relevant visual input and TMS conditions were evaluated through direct comparisons between TMS conditions. Consequently, interaction contrasts for (target present > target absent)_IPS (or Occ)_ > (target present > target absent)_Sham_ and (target absent > target present)_IPS (or Occ)_ > (target absent > target present)_Sham_ were estimated. Interaction effects between Occ- and IPS-TMS were directly evaluated in the same manner.

### Evaluation of phosphene perception

It is well established that in addition to being able to disrupt visual perception, TMS over the occipital cortex can elicit transient perceptions of light known as phosphenes. The ability to perceive phosphenes is quite variable across participants [44–46] and strongly depends on the amount of attention given to them [26, 36]. On the other hand, the intensity needed to elicit phosphenes is generally lower than the one necessary to induce effective suppression effects [26, 47, 48]. As phosphenes are elicited contra-laterally to the stimulated hemisphere, they could have potentially interfered with task performance [47–49]. To evaluate the effect of phosphenes in our study, at the end of the Occ-TMS session we asked participants whether they had perceived anything else apart from the standard visual display and requested them to draw what they saw. Only three participants reported having seen something, of which only two described image distortions near the placeholder. However, the reported distortions by one of these two participants were not specific to the vicinity of the placeholder but extended throughout the entire visual display comprising also the right visual field (i.e. ipsi-laterally to TMS stimulation), which suggests that the reported distortions were not ‘authentic’ phosphenes. The remaining participant that reported visual distortions adjacent to the placeholder also reported that these distortions were distinct from the task-relevant visual stimulus. Hence, in total it is unlikely that phosphene perception influenced our results on the group level both behaviourally and for the BOLD measurements.

### Eye monitoring (outside the scanner)

To ensure that the observed activation pattern did not result from eye movements, twitches, or startle effects, 8 additional participants (one left-handed; 2 male; mean age: 26.9 years; standard deviation: 5.3) took part in a supplementary TMS-psychophysics experiment outside the scanner performed with equivalent parameters and comparable durations. One experimental run was acquired per participant for each TMS condition. To account for the absence of the high-current filter used in the concurrent TMS-MRI setup [29], the TMS intensity was reduced to 63% of total output.

Horizontal and vertical eye movements were recorded using an iView XTM RED-III remote eyetracker system (SensoMotoric Instruments Inc., Needham/Boston, MA, USA) (50 Hz sampling rate). The eyetracking system was calibrated using a 13-point calibration. Eye position data were automatically corrected for blinks and converted to radial velocity.

For each trial condition the mean distance (degrees) from the fixation cross, the number of saccades (defined by a radial eye velocity threshold > 30°/s for a minimum of 60 ms duration and radial amplitude larger than 5°), and the proportion of blinks were computed for the entire trial duration separately for each individual condition and for baseline periods.

Across all participants, saccades were almost completely absent, precluding further statistical analyses for this index. For the two remaining indices we first evaluated whether they differed for activation trials and baseline periods. We therefore compared each index pooled (i.e. averaged) over all activation conditions with baseline periods in a paired t-test. The mean distance from the fixation cross was significantly greater during baseline periods (1.38° ± 0.44) than task trials (0.92° ± 0.25; paired t-test: t_*(7)*_ = -3.038, *p* = 0.019). We also observed a non-significant (t_*(7)*_ = -1.349, *p* = 0.22) increase in eye blinks for fixation baseline periods (1.79 ± 0.61) relative to activation trials (1.39 ± 0.54).

Second, to test for differences across individual task conditions, the two indices were independently entered into 2 (task-relevant visual input: V present, V absent) x 2 (auditory context: A present, A absent) x 3 (TMS: Occ, IPS, Sham) RM-ANOVAs. Importantly, neither of the two RM-ANOVAs revealed any significant main effects or interactions, thereby confirming that there was no significant difference in eye movements amongst our experimental conditions.

Collectively, these results demonstrate that differences in eye movements across task conditions are unlikely to account for activation differences across the eight task conditions. However, activation differences between task conditions and fixation baseline may result from eye movements that participants made during the fixation conditions when they recovered from the very demanding sustained attention task. Activations and deactivations relative to fixation baseline may in part be accounted for by eye movement confounds. Hence, the parameter estimate plots for the fMRI data should be interpreted only by comparing the eight task conditions, while the activation or deactivation relative to fixation should not be further interpreted.

## Results

### Behavioural data

In a sustained spatial attention paradigm, participants reported whether they had detected a visual target that was presented on 50% of the trials. For each participant, the behavioural indices were calculated for visual target present and visual target absent trials separately for each auditory context and TMS condition. Across participants’ mean (± SD) of % correct responses and reaction time data are summarized for each condition in Table 1.

**Table 1:** Behavioural responses averaged across participants (± SD).

*TMS Sites*	*% Correct*				*Reaction Times (ms)*			
	*A Present*		*A Absent*		*A Present*		*A Absent*	
	*V Present*	*V Absent*	*V Present*	*V Absent*	*V Present*	*V Absent*	*V Present*	*V Absent*
*IPS*	75 ± 21	98 ± 1	75 ± 25	98 ± 2	708 ± 75	692 ± 76	707 ± 73	711 ± 63
*Occ*	68 ± 24	99 ± 1	67 ± 25	98 ± 1	707 ± 90	700 ± 85	716 ± 100	720 ± 90
*Sham*	77 ± 13	99 ± 1	79 ± 11	99 ± 1	695 ± 88	677 ± 103	701 ± 91	698 ± 94

For % correct responses, a 2 (visual input: present vs. absent) x 2 (auditory context: present vs. absent) x 3 (IPS-TMS vs. Occ-IPS vs. Sham-TMS) RM-ANOVA revealed a significant main effect of visual input (*F*_*(1*,*7)*_ = 16.356; *p* = 0.005). This analysis did not reveal any other significant main effects (TMS: *F*_*(1*,*7)*_ = 1.418; *p* = 0.275; Auditory Context: *F*_*(1*,*7)*_ = 0.000; *p* = 0.988) nor interactions between the different factors (TMS and Auditory Context: *F*_*(1*,*7)*_ = 2.233; *p* = 0.144; TMS and Visual Input: *F*_*(1*,*7)*_ = 1.239; *p* = 0.320; Auditory Context and Visual Input: *F*_*(1*,*7)*_ = 0.120; *p* = 0.739; Interaction between the three: *F*_*(1*,*7)*_ = 1.598; *p* = 0.237). Participants missed about 25–30% of the targets, but showed nearly no false alarms. This confirmed that participants indeed placed a strict criterion for responding ‘target present’ in line with the accuracy instructions. None of the other main effects, 2-way or 3-way interactions were significant.

Median reaction times from each participant were entered in a 2 (visual: present vs. absent) x 2 (auditory context: present vs. absent) x 3 (IPS-TMS vs. Occ-IPS vs. Sham-TMS) RM-ANOVA that revealed only a significant interaction between auditory context and visual input (*F*_*(1*,*7)*_ = 9.000; *p* = 0.020). More specifically, auditory context shortened reaction times predominantly when the visual signal was absent, than when it was present. This suggests that the auditory signal served as a precise temporal cue for response preparation, when the target was not presented leading to faster ‘no’ responses. None of the main effects or other 2-way or 3-way interactions was significant (main effects of TMS: *F*_*(1*,*7)*_ = 0.418; *p* = 0.642, main effect of visual input: *F*_*(1*,*7)*_ = 0.342; *p* = 0.577; main effect of auditory context: *F*_*(1*,*7)*_ = 3.873; *p* = 0.090, interaction between the TMS and auditory context: *F*_*(1*,*7)*_ = 0.169; *p* = 0.842, interaction between TMS and visual input: *F*_*(1*,*7)*_ = 0.328; *p* = 0.683, interaction between the three factors: *F*_*(1*,*7)*_ = 0.622; *p* = 0.524).

Importantly, in line with previous concurrent TMS-fMRI studies [12, 50], our TMS manipulation did not elicit any behavioural changes in terms of % correct or reaction times. Further, additional analyses using d’ statistics from signal detection theory or participant-specific mean rather than median response times did not reveal any significant effect of TMS. Hence, our TMS manipulation functioned as a purely physiological perturbation method that allowed us to examine the influences of local perturbations on remote interconnected brain areas unconfounded by behavioural differences.

### Neuroimaging data

#### Main effects of task-relevant visual input

We evaluated the main effects of task-relevant visual input by pooling over auditory contexts and TMS conditions. Visual target presentation suppressed activations in the left precuneus, but this effect was only marginally significant when corrected for multiple comparisons within the entire brain (*p* = 0.09; [x = -14, y = -60, z = 42]; peak t-value: 4.91, number of voxels in cluster: 666). Further, we observed significant activations in the intraparietal sulcus (*p*_*IPS*_ = 0.012; [x = 32, y = -64, z = 48]; peak t-value: 10.40). Comparing visual target present relative to visual target absent did not reveal any significant effects.

#### Effects of TMS

We evaluated the effects of TMS by individually comparing IPS-TMS and Occ-TMS relative to Sham-TMS pooled over sensory conditions.

**Fig 2:**
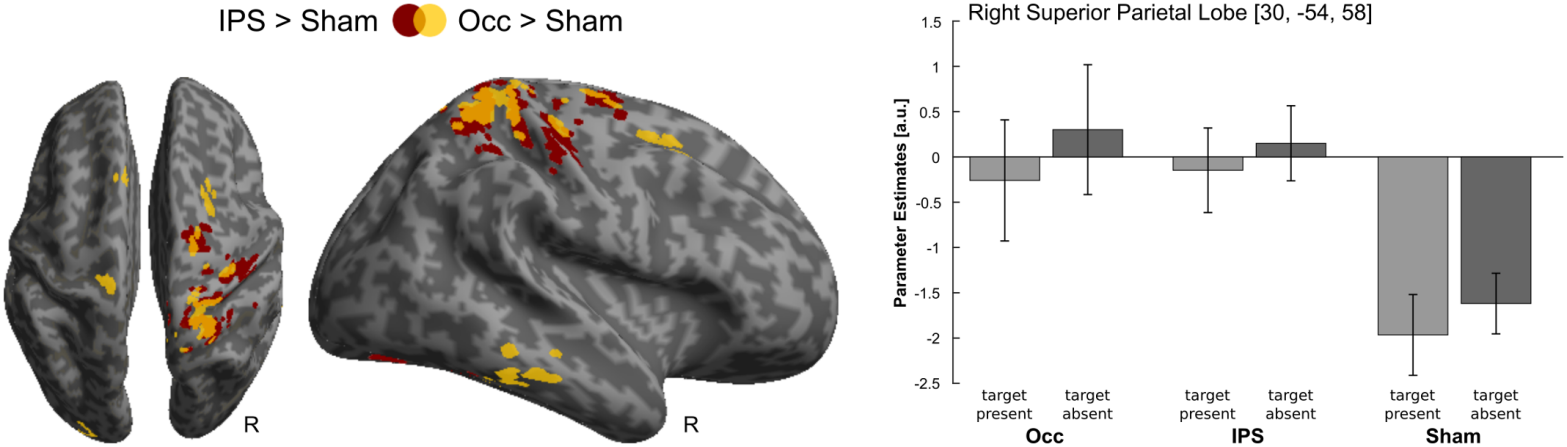
Main effects of TMS. (*left panel*) Activations induced by IPS- relative to Sham-TMS (red) and Occ- relative to Sham-TMS (yellow) are rendered on an inflated SPM template of the entire brain. For illustrational purposes only, effects are displayed at a height threshold of *p* = 0.01 uncorrected and an extent threshold of 100 voxels. (*right panel*) Parameter estimates (mean ± standard error of the mean) are displayed at the given peak coordinates within the parietal cortex. Parameter estimates are pooled (i.e. summed) over auditory contexts. The bar graphs represent the size of the effect in non-dimensional units (corresponding to % whole-brain mean).

As previously reported [15], the right parietal cortex showed increased activations for IPS- relative to Sham-TMS (*p*_*IPS*_ = 0.09; [x = 30, y = -54, z = 58]; peak t-value: 5.80; see also Fig 2). The opposite comparisons did not yield significant results. Likewise, comparing Occ-TMS with Sham-TMS did not result in any significant effects.

As shown in the parameter estimate plots (Fig 2), we observed only small (or even no) activations for task relative to fixation conditions during Occ- and IPS-TMS, but pronounced task-induced deactivations during Sham-TMS conditions. However, as previously argued [15], the comparison between task and fixation conditions should not be further interpreted, as this comparison may be confounded by differences in eye movements which are known to affect IPS activations. Importantly, there were no significant differences in eye movements between the different task or TMS conditions, so that the comparison between Occ, IPS and Sham-TMS and their interactions are not confounded by differences in eye movements and can therefore be interpreted.

#### State-dependent TMS effects: Interaction effects between visual input and TMS

Interaction effects between the task-relevant visual input and TMS were evaluated separately for (i) IPS-TMS vs. Sham-TMS and (ii) Occ-TMS vs. Sham-TMS.

The modulatory effect of IPS-TMS on visual processing was evaluated by testing for the interaction between IPS- vs. Sham-TMS and visual target present vs. absent. The results are in line with those reported in our previous communication, with a marginally significant interaction found in the right insula extending to the right temporal pole (*p* = 0.06; [x = 52, y = 6, z = -16], peak t-value:14.83; number of voxels in cluster: 794; for further discussion see [15]). The opposite interaction showed significant effects with the intraparietal sulcus (*p*_*IPS*_ = 0.012; [x = 44, y = -58, z = 42]; peak t-values: 10.45). We did not observe significant interaction effects between Occ- vs. Sham-TMS and visual target present vs. absent.

## Discussion

Classical fMRI studies in patients enable us to investigate the effect of permanent lesions on functional brain organization. Yet, variability in lesion location, extent and long-term functional reorganization often limit the interpretation of patient fMRI studies. Concurrent TMS-fMRI has been advocated as a transient perturbation method to provide complementary insights into the functional contributions of regions within a brain network. Yet, despite its potential, to our knowledge this is the first concurrent TMS-fMRI study that compared the effects of parietal and occipital TMS on visual target responses under sustained spatial attention in an attempt to ‘mimick’ neglect and hemianopia. Starting from the interpretational limitations and challenges of our own study we will provide guidance on how to optimize experimental design and TMS protocol when comparing TMS effects in primary sensory (e.g. visual) and higher order association areas (e.g. parietal), which differ profoundly in their computational principles [51], neural dynamics [52] and connectivity architecture.

In principle, TMS can induce four sorts of effects: i) neural effects locally under the coil, ii) remote neural effects via trans-synaptic spread of activation (measured as changes in effective connectivity in fMRI), iii) changes in behavioural performance and iv) physical side effects (e.g. clicking noise, tickling sensation), which again are associated with confounding neural and behavioural changes. Concurrent TMS-fMRI studies enable us to infer TMS-induced changes in functional network architecture or remote activations (i.e. type ii effects) unconfounded by any of the other effects. Thus, in our study we focused on TMS induced perturbations of parietal and occipital cortices controlled for behavioural effects [53]. In contrast to the careful behavioural matching, our study was less successful in matching the local neural effects for IPS and occipital TMS perturbation. While IPS-TMS increased activations relative to Sham-TMS in parietal cortices underneath the coil, Occ-TMS did not induce TMS-effects on the BOLD-response directly underneath the coil. The absence of direct activations underneath the coil is not unusual in concurrent TMS-fMRI studies [14, 28, 32, 50, 54–56]. Yet, it limits the interpretation of differences between Occ and IPS-TMS perturbation on the neural processing systems.

In the following, we will discuss and provide guidance on how future studies should optimize experimental design and TMS protocol to match local neural and behavioural effects and thereby enable strong interpretations.

### Optimizing experimental design and TMS protocol with respect to local neural effects

First, to promote effective Occ- and IPS-TMS neural perturbations the experimental design should elicit reliable neural activity in both primary and higher-order association areas. In our current study we employed a sustained spatial attention paradigm that presented a small visual stimulus with the visual contrast fine-tuned individually for each participant to obtain 70% detection rate (for studies using a similar approach see IPS-TMS: [17], or Occ-TMS: [18, 24, 26, 36, 47, 57–61]). This low contrast visual stimulus placed high attentional demands thereby maximizing our chances to reveal direct IPS-TMS effects on neural responses in parietal cortices. Yet, the visual stimulus did not induce reliable visual activations in striate or extrastriate cortices even in the absence of TMS. Thus, our paradigm may have been sub-optimal for unravelling direct or modulatory effects of Occ-TMS on visual evoked activations. To maximize TMS effects simultaneously on bottom-up driven visual activations and direct and top-down modulatory effects of IPS, future studies should therefore combine high contrast stimuli to drive visual processing with a demanding attentional task to tax attentional processing [12, 37].

Second, concurrent TMS-fMRI studies need to optimize the TMS protocol for perturbing primary sensory and higher order association areas that differ profoundly in their temporal scales of neural dynamics [62–64]. While primary sensory areas show brief transient responses to sensory inputs, association areas exhibit more sustained activity by integrating inputs over time. In principle, one could use two different TMS protocols. For instance, following the TMS protocols from previous behavioural studies one could apply a single TMS pulse at a specific latency in occipital cortices [25, 35, 36], but 10 Hz bursts of 4 TMS pulses in parietal cortices [24, 31, 32, 34]. However, the use of different TMS protocols would introduce interpretational limitations, because the TMS BOLD-effects are known to scale with the number of TMS pulses applied [65]. Therefore, in the current study we used one TMS protocol that optimized the timing of the first TMS pulse for occipital regions, but the number of TMS pulses according to the parietal area exhibiting longer sustained neural activity (i.e. 4 TMS pulses at 10 Hz).

Nevertheless, based on the absence of a TMS effect on the BOLD-response locally under the coil for Occ-TMS, one may argue that the protocol was not equally effective for Occ- and IPS-TMS. Yet, in the light of variability in neurovascular coupling across regions we would caution against using the BOLD-response as the gold standard parameter to match TMS effectiveness across regions. Instead, we would suggest applying the same basic TMS protocol, but adjust TMS intensity individually to each TMS site. For some target regions it may be feasible to adjust TMS intensity in terms of the phosphene or motor threshold. In others regions, TMS intensity is adjusted individually to each TMS site based on published scaling factors that account for differences in coil-cortex distances between the cortical sites [66–69] or even based on modelling of electromagnetic fields [70, 71], which additionally accounts for the impact of gyrifications. Further, to account for the fact that TMS effects are strongly context-sensitive and state-dependent, one may also characterize the relation between local BOLD effects on TMS intensity in input-output curves during initial pilot experiments. Finally, the use of new RF coil designs will likely also prove advantageous, as they are more sensitive directly underneath the TMS coil [72].

### Optimizing experimental design and TMS with respect to behavioural effects

Matching TMS in term of local neural effectiveness could potentially lead to different behavioural effects across TMS sites. For instance, TMS to primary visual areas may impact visual detection more strongly than TMS to parietal areas. As previously discussed in the context of patient fMRI studies [73, 74], these differences in behavioural effects limit the interpretation of activation differences across conditions. For instance, in the most extreme case, participants will not respond to visual stimuli they are not aware of during Occ-stimulation, leading to reduced neural activations in widespread systems including prefrontal and occipito-temporal cortices associated with visual perception, response selection and motor response. To avoid these behavioural confounds we should either adjust the experimental paradigm or the TMS intensity. For instance, in studies of visual perception we could increase the stimulus intensity or focus on implicit processing where participants do not explicitly respond to the dimension of interest [75]. In the current paradigm our threshold stimuli allowed us to characterize TMS induced differences in neural activations in the absence of behavioural effects. Yet, the absence of behavioural effects does not provide direct insights into the functional relevance of the TMS-induced activation changes. Therefore, we would suggest that future experiments should combine conditions where neural effects are or are not associated with behavioural changes. For instance, to compare TMS to parietal and primary visual areas experimental designs may i) present threshold and strong supra-threshold visual stimuli, ii) focus on explicit and implicit stimulus processing [75] or iii) include two levels, i.e. sub- and supra-threshold, of TMS intensity.

### Optimizing TMS to control for physical TMS-side effects

In addition to true neural TMS effects, studies focusing on visual, auditory or somatosensory processing need to control for somatosensory and auditory TMS side effects across different TMS stimulation sites. Previous studies have suggested that low intensity TMS may act as a control condition. Yet, a recent study by Leitão et al. [14] demonstrated that low intensity TMS is not an appropriate control condition, as it is not matched in terms of auditory amplitude to high TMS. This does not only affect studies in the auditory system, but also somatosensory and visual processing because of crossmodal interactions such as crossmodal deactivations [76, 77]. Indeed, high intensity TMS leads to greater deactivations in primary and higher order visual system than low intensity TMS, thereby confounding the interpretation of nearly any statistical comparison [14]. To resolve these interpretational ambiguities, we have developed a Sham condition, where we fixed 2 cm thick plastic plates between the TMS coil and the skull. Given the quadratic decay of the TMS-induced magnetic field, this Sham condition precluded the effects of direct brain stimulation. Yet, the Sham-TMS condition tightly controlled for the TMS side effects such as the TMS-noise and feelings of vibrations. Indeed, comparing IPS-TMS or Occ-TMS with Sham-TMS did not reveal significant activations in the auditory cortex. However, it is worth mentioning that this Sham condition has limited effectiveness in studies that stimulate positions for which the side effects result mainly from nerve or muscle stimulation rather than vibrations, such as when TMS is applied to over temporal muscles or some more lateral and inferior regions of the occipital cortex (M. occipitalis).

### Conclusions

To compare TMS perturbations to low level sensory and higher order association areas we would suggest matching the TMS protocols in terms of number and timing of pulses, but adjust TMS intensity individually across sites to control for differences in coil-cortex distances. Further, the experimental paradigm should be designed to elicit strong activations in both neural systems that one hopes to perturb. It should ideally include one condition that is associated with behavioural TMS effects to assess regional contributions to behavioural performance and one condition that is not associated with behavioural TMS effects to allow assessment of regional neural contributions and effective connectivity between regions unconfounded by differences in behavioural performance.

## Supporting information

S1 File Aggregate data. Aggregate data containing behavioural data and beta values used in the parameter plots of Fig 2.
(ZIP)
